# Does Adherence to Treatment Guidelines from the Ghailane–Gille Classification for Degenerative Spondylolisthesis of the Lumbar Spine Impact Surgical Outcomes? A Match–Mismatch Study

**DOI:** 10.3390/jcm14062041

**Published:** 2025-03-17

**Authors:** Ghailane Soufiane, Campana Matthieu, Gille Olivier, Bouloussa Houssam, Jacquemin Clément, Castelain Jean Etienne, Challier Vincent

**Affiliations:** 1 Department of Spinal Surgery Unit, Hôpital Privé Francheville, 24000 Périgueux, France; drcampana@hpdf.fr (C.M.); clement.jacquemin@hpdf.fr (J.C.); drcastelain@hpdf.fr (C.J.E.); drchallier@hpdf.fr (C.V.); 2 Department of Spinal Surgery Unit 1, Université de Bordeaux, Bordeaux University Hospital, C.H.U. Tripode Pellegrin, Place Amélie Raba Léon, 33076 Bordeaux, France; olivier.gille@chu-bordeaux.fr; 3 Department of Orthopaedic Surgery, University of Missouri-Kansas City, 2301 Holmes Street, Kansas City, MO 64108, USA; houssam.bouloussa@gmail.com

**Keywords:** degenerative spondylolisthesis of the lumbar spine, sagittal alignment, ASA

## Abstract

**Background/Objectives**: satisfactory sagittal alignment when treating degenerative spondylolisthesis of the lumbar spine (DSLS) may produce better clinical and radiographic outcomes compared to treatment focused solely on isolated segments when indicated. Ghailane et al. proposed a treatment guideline based on their classification system. The aim of this study was to investigate the impact of adherence to Ghailane–Gille (GG) treatment guidelines on surgical outcomes in patients with DSLS. **Methods**: A monocentric retrospective cohort analysis was performed from 2021 to September 2024. Data were collected from patients treated for DSLS, covering the period from baseline to one-year follow-up. Patients were divided into two groups based on GG treatment guidelines: the “Match group” (patients who underwent surgery following GG guidelines) and the “Mismatch group” (patients who did not adhere to these guidelines). Preoperative and postoperative clinical outcomes, patient satisfaction, and operative parameters were collected and compared between groups. **Results**: A total of 80 patients were enrolled, with 52 in the Match group and 28 in the Mismatch group. At baseline, the Oswestry Disability Index (ODI) score demonstrated significant variation among classification subtypes and a positive correlation. The Match group exhibited a significant reduction in ODI scores one year postoperatively and maintained high levels of satisfaction; no significant intraoperative differences were noted. Additionally, patients in the Mismatch group were more frequently classified as *American Society of Anesthesiologists* (*ASA*) III compared to the Match group (70% vs. 30%), suggesting clinicians’ hesitance to fully implement GG guidelines in aggressive treatment strategies for those patients. **Conclusions**: Adhering to the GG treatment guidelines for restoring sagittal alignment in DSLS patients is associated with decreased ODI scores regardless of age, ensuring patient satisfaction at one-year follow-up. This approach could potentially benefit ASA III patients as well.

## 1. Introduction

Degenerative spondylolisthesis of the lumbar spine (DSLS) is a prevalent cause of low back pain, leg pain, neurogenic claudication, and sagittal malalignment [1,2]. Surgical management is indicated when maximal conservative treatment has failed. Various options may be discussed, including decompression alone, dynamic stabilization, or fusion with or without deformity correction [3].

Several classification systems exist for DSLS. The most commonly used is the Meyerding classification, which describes five grades based on the degree of vertebral translation [4]. Unfortunately, it is purely radiographically descriptive as slip severity was not demonstrated to correlate with clinical outcomes [5]. Also, the clinical and radiographic degenerative spondylolisthesis (CARDS) classification introduced in 2014 presented four main radiographic types based on three radiographic features (disc height, vertebral translation, and segmental alignment) with a modifier for leg pain was the first to incorporate a clinical component. However, there is no treatment recommendation or consideration given to spinal alignment [6].

The degenerative spondylolisthesis instability classification (DSIC) [7] was proposed in 2015 and provided guidance based on the assessment of stability. The parameters included are back pain, restabilization signs (disc height loss, osteophyte formation, and endplate sclerosis), facet joint effusion, disc angle, and magnitude of dynamic translation. The three defined types and their association with treatment recommendation are the following: Type I (stable): decompression alone; Type II (potentially unstable): decompression with posterior fusion; Type III (unstable): decompression and posterior fusion with inter-body placement. This classification emphasizes stability and back pain as a main clinical parameter while the presence of lumbar radiculopathy and its etiology is not tackled.

The Kulkarni scoring system (2020) aimed to rationalize the need for fusion or not for patients with DSLS [8]. This more complex and the weighted scoring system incorporates a clinical, radiographic, and subjective surgical component: mechanical back pain (2), age < 70 years (1), high-demand activity (1), segmental kyphosis (1.5), Dynamic translation > 2 mm (1), disc height > 50% of adjacent level (1), bilateral facet effusion on MRI (1), sagittal facet orientation (1), and feasibility of performing a decompression without compromising stability (1.5). The weight of each parameter is debatable (back pain is vague, not defined as positional, and only adds 2 points) and no consideration for spinal alignment is provided. The authors recommend fusion when the sum exceeds 5.5.

The correlation between DSLS and spinopelvic alignment has been illustrated by several authors [9,10,11,12]. In 2007, Barrey et al. demonstrated that patients with high pelvic incidence are predisposed to develop DSLS [13]. Postoperative alignment was associated with Health-Related Quality of Life Scores (HRQOL) in a few studies [14], despite Karim et al. not finding such correlation preoperatively [15]. Alignment should be a consideration in surgical planning [16].

More recently, Rangwalla et al. proposed the UCSF classification system for DSLS [17], which is an attempt to synthesize all previous classifications and provide treatment recommendations based on four components: (1) segmental dynamic instability, (2) location of spinal stenosis, (3) sagittal alignment, and (4) primary clinical presentation. The location of spinal stenosis represents a novel addition while the other parameters were previously described in other classifications. Of note, the incorporation of both back pain and leg pain in the same classification had not been performed before. The authors demonstrated that this classification was associated with high intra and inter-observer reliability. This classification is not clinically validated and its influence on treatment has not been tested to date. While the authors described treatment patterns, no formal algorithm was described.

Ghailane and Gille (GG) proposed and validated a classification system for DSLS based on sagittal alignment (SA). Three types were described according to the SRS-Schwab classification (GG classification). Type 1 corresponds to a harmonious and aligned spine, Type 2 corresponds to compensated spinal malalignment, and Type 3 corresponds to altered global sagittal alignment. Treatment guidelines were presented according to this classification, aiding surgeons in enhancing their preoperative DSLS analysis [18,19]. Treatment recommendations are as follows: Type 1 requires a segmental strategy consisting of treating the spondylolisthesis level with decompression, possibly with dynamic stabilization or a one-level fusion. Types 2 and 3 necessitate regional or global correction to address the PI-LL mismatch and provide a harmonious distribution of lumbar lordosis. This study aimed to investigate the impact of adhering to GG treatment guidelines on short- and mid-term surgical outcomes in DSLS.

## 2. Materials and Methods

### 2.1. Study Design and Population

A single-center retrospective study was conducted involving patients diagnosed with degenerative spondylolisthesis of the lumbar spine (DSLS) who underwent surgery between 2021 and September 2024.

The inclusion criteria were as follows:

1. Age > 18 years;
2. Surgical treatment for DSLS;
3. Complete patient-reported outcomes, including the following:
  ○ Demographic information: gender, age, Body Mass Index (BMI), smoking status, and comorbidities;
  ○ Health-Related Quality of Life Scores (HRQOLS): Oswestry Disability Index (ODI), visual analog scales for back pain (BP-VAS), and leg pain (LP-VAS);
  ○ Radiological parameters: slippage level, segmental lordosis (SL), lumbar lordosis (LL), pelvic incidence (PI), pelvic tilt (PT), PI-LL mismatch, L4-S1 distribution, and sagittal vertical axis (SVA).

Preoperative and one-year follow-up postoperative patient-reported outcomes and radiographic measurements were collected and analyzed. All patients included in this study provided informed consent for the extraction and analysis of their data prior to any study procedures.

**Figure 1:**
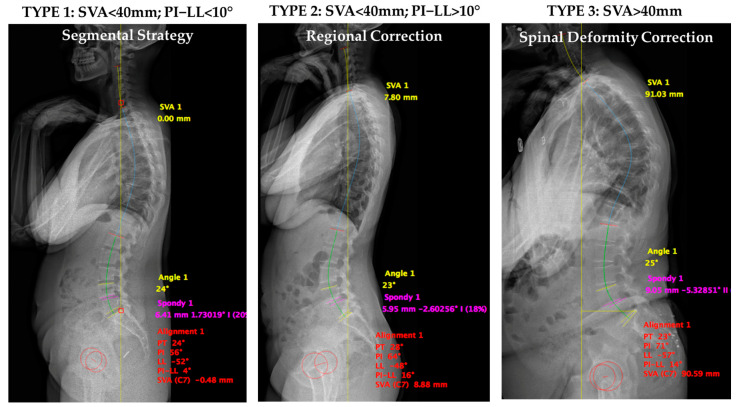
Figure introduction. Abbreviations: SVA; Sagittal Vertical Axis; PI; pelvic incidence; LL: lumbar lordosis.

Preoperative patients were classified into three types according to the GG classification system. For reference (Figure 1),

- Type 1 corresponds to a harmonious and aligned spine: SVA < 40 mm, PI-LL < 10°;
- Type 2 corresponds to compensated spinal malalignment: SVA < 40 mm, PI-LL > 10°, with thoracic compensation (Type 2A: PT < 25°) or pelvic compensation (Type 2B: PT > 25°);
- Type 3 corresponds to altered global sagittal alignment: SVA > 40 mm.

Following surgical planning, all patients were classified into two independent groups: the “Match” group, which included patients whose DSLS surgery adhered to GG guideline recommendations, and the “Mismatch” group, which included patients whose DSLS surgery did not adhere to GG guideline recommendations (referred to as rule-breakers).

### 2.2. Statistical Analysis

Descriptive statistics were calculated as means and standard deviations (SD) or frequencies and percentages. Differences were considered statistically significant at *p* < 0.05. Analyses were performed using GraphPad Prism 8.0 statistical software (GraphPad Software, La Jolla, CA, USA). *t*-tests and Mann–Whitney U tests were utilized for continuous variables, guided by normality analysis. Spearman’s correlation test was employed for two ranked variables, while Fisher’s exact test was used for contingency variables.

## 3. Results

### 3.1. Patient Cohort

A total of 80 patients met the inclusion criteria, comprising 59 females (73.75%) and 21 males (26.25%). The mean age of the overall cohort was 68.8 ± 9.9 years. The levels affected were as follows: L4-L5: *n* = 62 (77.5%), L3-L4: *n* = 8 (10%), L5-S1: *n* = 2 (2.5%), and multiple levels: *n* = 8 (10%). During surgery, no major complications were recorded.

Preoperatively, the distribution of patients according to the GG classification system was as follows: Type 1: *n* = 47 (58.75%), Type 2: *n* = 10 (12.5%), and Type 3: *n* = 23 (28.75%). Table 1 summarizes demographic and clinical data by subtypes.

### 3.2. Baseline Characteristics

**Figure 2:**
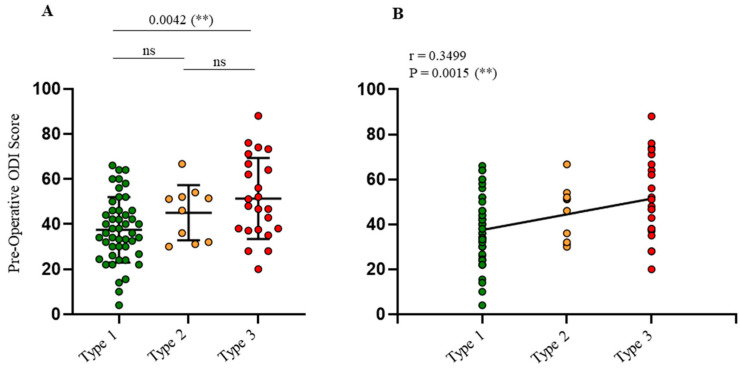
Preoperative ODI score distribution following GG classification subtypes. Overall, there were 80 DSLS patients subdivided in three types (Type 3 in red plot; *n* = 23—Type 2 in orange plot: *n* = 10; and Type 1 in green plot: *n* = 47) of preoperative ODI distribution (**A**) and correlation (**B**) are represented. (ns) correspond to not significant and (**) to *p* < 0.01.

Concerning baseline demographical data, no significant differences for gender, Body Mass Index (BMI), or smoking status were observed among the various types (Table 1). A significantly higher mean age was identified in Type 3 compared to Type 1 (*p* = 0.05), and the ODI was also significantly higher in Type 3 compared to Type 1 (*p* = 0.0042). A positive correlation between ODI scores and GG classification types was established (Figure 2A,B). No significant differences were observed in leg pain or back pain VAS scores among the groups.

In terms of preoperative radiographic parameters, Type 3 demonstrated moderate to marked deformity, exhibiting significant differences in specific sagittal and spinopelvic parameters, including pelvic tilt (PT), pelvic incidence (PI), lumbar lordosis (LL), PI-LL, and sagittal vertical axis (SVA) when compared to Types 1 and 2 (Table 2). No significant differences in radiographic findings were observed between Types 1 and 2.

### 3.3. Operative Details

Associations between surgical procedure characteristics—including estimated blood loss (cc), operative time (minutes), hospital length of stay (days), and surgical approaches—were assessed. No significant differences were observed between GG classification types, except for a higher percentage of ASA III classifications in Type 3 compared to Types 1 and 2. A low rate of early complications (within 30 days postoperatively) was noted, occurring only in the GG subtype 1 group. Evaluation of late complications revealed one minor complication in subtype 2 (soft tissue irritation opposite the scar) and one major complication in subtype 3 (L4-S1 arthrodesis revision due to adjacent segment syndrome), which required reoperation. Detailed operative characteristics by type are presented in Table 3.

### 3.4. Clinical Outcomes Following MATCH or MISMATCH Distribution

One year after surgery, patients were categorized into two groups: “MATCH” (*n* = 52) and “MISMATCH” (*n* = 28). The MATCH group consisted of patients whose surgical interventions adhered to GG classification guidelines, aiming to restore or maintain adequate sagittal alignment. In contrast, the MISMATCH group comprised patients whose treatments did not align with the GG classification recommendations, focusing solely on the affected vertebral level without addressing sagittal alignment. Post-operative radiographic parameters are presented in Supplemental Tables S1 and S2.

All patients in all groups benefited from the same rehabilitation program at our center, including physical therapy and core strengthening sessions.

The MATCH group exhibited significantly improved sagittal alignment parameters compared to the MISMATCH group at one-year follow-up. Postoperatively, the ODI for the MATCH group was significantly lower than that of the MISMATCH group (18.9 ± 12.9 vs. 29.4 ± 18.9; *p* = 0.0121) (Table 4).

No significant differences in leg pain (LP-VAS) or back pain (BP-VAS) scores were identified between the two groups. Patient satisfaction rates were comparable, with 60% to 70% of patients in both groups reporting satisfaction to very satisfaction with their outcomes. Finally, less than half the patients in both groups used analgesic drugs, among which 65 to 70% used acetaminophen and/or NSAIDs.

**Figure 3:**
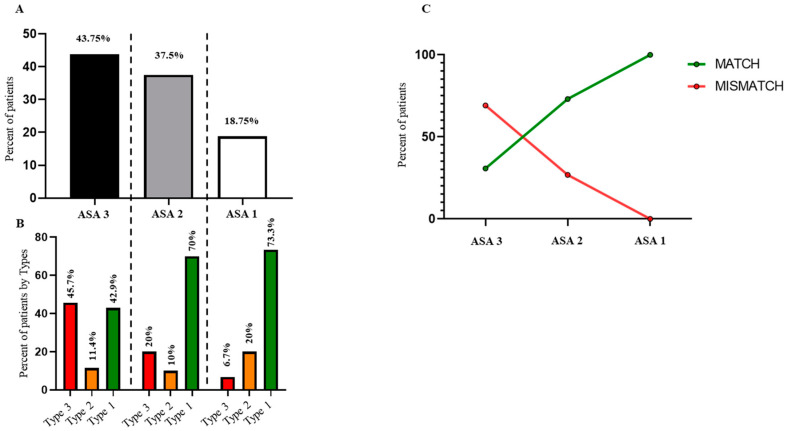
Preoperative ASA score distribution within postoperative MATCH or MISMATCH groups. All DSLS patients (**A**), preoperative subtypes (**B**), ASA score distribution and relationship between preoperative ASA score on post-operative matching versus mismatching groups (**C**).

Of note, 45.7% of patients classified preoperatively as ASA III were found in Type 3 and 42.9% in Type 1, while 73.3% of patients with an ASA I classification were categorized into Type 1 (Figure 3A,B). Additionally, there was an observable trend toward lower ASA scores in the MATCH group compared to the MISMATCH group (Figure 3C).

## 4. Discussion

DSLS is a condition that appears to exist at the intersection of degenerative processes and spinal deformities. The GG classification illustrates the progression of malalignment severity among the three types [18,19]. Multiple studies have reported that spinopelvic sagittal malalignment plays a significant role in adult spinal deformities [20,21,22]. In the context of DSLS, evidence supporting the relationship between health-related quality of life scores (HRQOLS) and alignment parameters is lacking. At baseline, the three types demonstrated increased ODI scores, with significant differences between types 1 and 3, as well as a significant positive correlation among the types. These findings support previous research published in a 2017 study analyzing another DSLS cohort [18]. In 2022, Karim et al. stated that sagittal and spinopelvic malalignment were not associated with HRQOL in patients with DSLS at baseline [15]. The discrepancy with current results may be attributed to different methodologies for defining the groups. In our model, preoperative patient classification was based on PI-LL and SVA (40 mm), whereas Karim et al. evaluated their groups using PI-LL and pelvic tilt (PT).

In our experience, a thorough clinical examination combined with detailed analysis of local, regional, and global alignment through standing X-rays and MRI imaging is essential to maximize the chances of surgical success. In this study, all patients exhibited significant back pain (ODI > 40) and baseline LP-VAS scores indicative of a severely diminished quality of life, which justified the need for surgical intervention.

The decision-making process shows considerable variability within the spine community, especially in the management of DSLS. To date, there remains no consensus regarding surgical indications and techniques. While surgical treatment appears to yield better outcomes than conservative strategies [23], the optimal surgical technique remains a topic of controversy [24,25]. In 2014, Keppler et al. proposed a four-subtype classification system focusing on local radiological parameters, including disc space, local kyphosis, translation, and leg pain. Near-perfect agreement was observed regarding inter-observer and intra-observer reliability, although no analysis of preoperative to postoperative clinical outcomes was conducted [26]. The purpose of the current study was to evaluate the impact of local, regional, and global alignment on the decision-making process. The MATCH classification was defined retrospectively based on harmonious conservation and/or improvement in sagittal alignment, whereas the MISMATCH group was defined by deterioration in sagittal alignment. ASA III patients were significantly more prevalent in Type 3, illustrating the relationship between severe malalignment and frailty, as reported by Passias et al. [26]. Additionally, ASA III patients were also predominant in the MISMATCH group, indicating that frailty influenced the decision-making process. An aggressive strategy aimed at restoring harmonious alignment may not be compatible with the surgeon’s mindset.

A fair correlation between GG types and preoperative ODI scores was observed in the current cohort. As highlighted in several studies [2,27], the predominance of female patients was evident. Most importantly, we demonstrated clinical benefits one year postoperatively by achieving or maintaining sagittal alignment goals in accordance with the GG classification for surgical treatment of DSLS. Indeed, MATCH patients exhibited better postoperative ODI scores (18.9 ± 12.9). Furthermore, for Type I degenerative lumbar spondylolisthesis, the minimally clinically important difference (MCID) was achieved (14.3 points) in the MATCH group [28].

Throughout the analysis, the authors encountered new challenges. Some patients initially classified as Type 3 might have been more appropriately classified as Type 1 according to the GG classification. Distinguishing between DSLS associated with compensatory mechanisms and a rigid degenerative condition can be complex [29]. In cases where compensatory mechanisms remain functional, the spine may display a degree of imbalance that the body can counterbalance, resulting in a dynamic and fluctuating clinical presentation. A straightforward approach involving decompression or single-level fusion may correct the sagittal imbalance. However, in a rigid degenerative condition, the spine loses the ability to compensate, leading to a fixed sagittal imbalance that is unresponsive to postural adjustments. The effectiveness of these interventions can vary based on individual patient characteristics and underlying pathologies [30]. This type of rigid imbalance often proves to be more debilitating, as the absence of compensatory ability results in persistent and pronounced misalignment that significantly impacts the patient’s overall posture and function. Distinguishing between these two scenarios is critical for effective treatment planning, as therapeutic approaches may vary considerably based on the underlying pathology. A comprehensive understanding of sagittal imbalance necessitates a thorough physical examination and dynamic assessment of the patient, along with detailed MRI analysis. While EOS imaging provides valuable insight into overall alignment, it is inadequate alone to guide surgical planning. A holistic approach that incorporates both physical and dynamic evaluations alongside advanced imaging techniques is essential for informed decision-making regarding treatment.

In spinal surgery, the importance of meticulous operative planning cannot be overstated, particularly when addressing conditions such as sagittal imbalance [31,32]. Proper preoperative planning facilitates a comprehensive approach that anticipates potential challenges during the intervention [33]. However, it is essential to emphasize that the successful execution of the planned procedure is paramount; clinical outcomes are heavily dependent on the adequacy of the surgical performance. Even with a well-structured plan, the actual execution of the surgery plays a more critical role in determining patient outcomes than the planning itself. For instance, some patients categorized as Type 3 may continue to be classified as such if the surgery, despite careful planning, is poorly executed, even if the goal was to restore them to Type 1. Therefore, both planning and execution must be regarded as integral components of a successful surgical intervention.

Several attempts have been made to establish therapeutic guidelines for degenerative spondylolisthesis. Mannion et al. found that the “appropriate use criteria”, defined by the North American Spine Society (NASS) [34], could predict clinical improvement in a controlled multicenter prospective study. However, it remains unclear which patients experience a greater treatment effect from surgery compared to non-surgical management [35].

Furthermore, validation of the CARDS classification as a treatment guideline has been challenging due to difficulties in controlling for confounding variables. The primary limitation is the absence of randomization, as surgeons likely considered additional factors beyond the CARDS classification when determining their surgical strategy [6].

Additionally, comparisons between the DSIC and CARDS classifications showed that clinical outcomes for uninstrumented and instrumented surgical techniques were generally similar across categories. However, these findings were inconclusive as a validation strategy for treatment guidelines due to the lack of randomization. Notably, the number of patients in the CARDS D (*n* = 15) and DSIC 3 (*n* = 17) uninstrumented groups was low, likely reflecting surgeons’ preference to avoid uninstrumented techniques in these patients. These studies highlight the critical need for high-quality methodological approaches to assess the impact of adherence to treatment guidelines [36]. The key issue is the likely reluctance of both surgeons and patients to accept significantly different surgical strategies based solely on randomization.

This study has several limitations. First, it is a single-center retrospective study with a small patient sample and a relatively short one-year follow-up period, which may not fully capture potential long-term differences in hardware failure, adjacent segment degeneration, or spinal alignment progression. Additional studies with larger cohorts, diverse geographical representation, and extended follow-up durations are needed.

Second, patients in the Mismatch group may have been selected for less aggressive treatment due to comorbidities or anatomical and technical considerations. The lack of randomization is a key limitation. No classification system can substitute for a thorough informed consent process, which includes detailed discussions of risks and benefits. Patients and surgeons may opt against more extensive surgery due to concerns about postoperative complications, prioritizing immediate safety over potential long-term quality-of-life improvements. Nevertheless, understanding the outcomes associated with deviations from classification-based recommendations remains important.

Third, our study population consisted of relatively older adults (mean age 68.8 years), which may limit external validity. Fourth, all patients in this study underwent surgical treatment; future research comparing surgical and conservative management strategies could provide valuable insights.

Lastly, the use of ODI is limited by its subjective assessment of disability [37]. In lieu of ODI, objective assessment tools such as the World Health Organization Disability Assessment Schedule 2.0 (WHODAS 2.0) emphasize an individual’s level of functioning in major life domains. This aligns with the concepts of the International Classification of Functioning, Disability, and Health (ICF) (World Health Organization. Measuring health and disability: manual for World Health Organization (WHO) Disability Assessment Schedule 2.0 (WHODAS 2.0) [38]. As the goal of surgery is not only to improve quality of life but also functioning, postoperative clinical improvement assessment should be facilitated by the use of this validated questionnaire [39].

Additionally, it is essential to analyze preoperative MRI findings to assess the influence of central or foraminal stenosis on sagittal imbalance, as this could significantly impact treatment decisions and patient outcomes. Our proposed therapeutic recommendations aim to assist surgeons in their decision-making and surgical planning. However, these recommendations must take into account intrinsic patient parameters, such as comorbidities, frailty, and the patient’s willingness to adhere to the treatment, as well as extrinsic factors, including an analysis of functional complaints and compressive components observed on MRI, in addition to sagittal balance, recognizing that sagittal balance is just one symptom among many.

## 5. Conclusions

The present study demonstrates that sagittal and spinopelvic malalignment are associated with preoperative clinical outcomes. Surgical planning and execution in accordance with the GG classification can enhance clinical outcomes and sustain patient satisfaction in individuals over 65 years old with degenerative spondylolisthesis of the lumbar spine (DSLS). However, it is important to note that management remains complex and must consider several factors, including the patient’s frailty and their specific functional complaints. Further research needs to be undertaken to fully assess the outcomes of the adherence to our treatment guidelines beyond two years.

## 6. Patents

No patents resulting from the work reported in this manuscript.

## Figures and Tables

**Table 1:** Demographical and clinical characteristics. Abbreviations: SD: Standard Deviation; ODI: Oswestry Disability Index; VAS: Visual Analog Scales; DSLS: Degenerative Spondylolisthesis of the Lumbar Spine. (**) correspond to *p* < 0.01.

	ALL PATIENTS (*N* = 80)	TYPE 3 (*N* = 23)	TYPE 2 (*N* = 10)	TYPE 1 (*N* = 47)	TYPE 3 vs. 2	TYPE 3 vs. 1	TYPE 2 vs. 1
	Mean (±SD)				*p*-Value		
Gender							
Women	59 (73.75%)	20 (86.95%)	7 (70%)	32 (68.08%)	0.3364	0.1446	>0.9999
Men	21 (26.25%)	3 (13.05%)	3 (30%)	15 (31.92%)			
Age (mean, ±SD)	68.8 ± 9.9	72.7 ± 9.2	71.7 ± 8.8	66.8 ± 9.9	0.9931	0.0536	0.3905
Body Mass Index, kg/m^2^	27.8 ± 4.9	27.6 ± 5.1	25.5 ± 3.6	28.5 ± 5	0.6760	0.9015	0.3147
Smoker (%)	14 (17.5%)	4 (17.4%)	3 (30%)	7 (14.9%)	0.6456	>0.9999	0.3568
ODI	42.4 ± 16.4	51.3 ± 17.9	45 ± 12.2	37.5 ± 16.4	0.7236	0.0042 (**)	0.5187
VAS Back (Scale 0–10)	7.3 ± 1.9	8 ± 1.5	6.7 ± 2.5	7.1 ± 1.9	0.3153	0.3091	0.9300
VAS Legs (Scale 0–10)	7.2 ± 1.9	6.9 ± 2.3	7.5 ± 1	7.3 ± 1.9	0.8556	0.8233	0.9954
DSLS Diagnosis							
L3L4	8 (10%)	3 (13.1%)	1 (10%)	4 (8.5%)			
L4L5	62 (77.5%)	13 (56.5%)	9 (90%)	40 (85.1%)			
L5S1	2 (2.5%)	1 (4.3%)	0	1 (2.1%)			
Multiple	8 (10%)	6 (26.1%)	0	2 (4.3%)			

**Table 2:** Preoperative Spinal Alignment Parameters. Abbreviations: PT: Pelvic Tilt; PI: Pelvic Incidence; LL: Lumbar Lordosis; SL: Segmental Lordosis; SVA: Sagittal Vertical Axis. Bold numbers show significant *p*-value and (*) for *p* < 0.05, (**) for *p* < 0.01, (***) for *p* < 0.001 and ****p < 0.0001.

	ALL PATIENTS (*N* = 80)	TYPE 3 (*N* = 23)	TYPE 2 (*N* = 10)	TYPE 1 (*N* = 47)	TYPE 3 vs. 2	TYPE 3 vs. 1	TYPE 2 vs. 1
	Mean (±SD)				*p* Value		
Pre PT (°)	22.6 ± 7.4	27.6 ± 8.8	21.8 ± 6.6	19.6 ± 6.9	0.0392 (*)	<0.0001 (****)	0.2462
Pre PI (°)	60.4 ± 11.4	66.6 ± 13	60.5 ± 12.3	57.4 ± 9.2	0.1764	0.0007 (***)	0.4522
Pre PI-LL (°)	6.2 ± 14.1	17.3 ± 16.3	2.3 ± 10.7	1.6 ± 10.2	0.0095 (**)	<0.0001 (****)	0.8484
Pre SL (°)	54.2 ± 12	4.3 ± 3.2	5.4 ± 3.6	7 ± 4.2	0.4344	0.0071 (**)	0.1931
Pre LL (°)	54.2 ± 13	49.3 ± 15.1	58.2 ± 14.1	55.8 ± 11.1	0.2097	0.0861	0.8565
Pre L4S1 (°)	30.4 ± 10.3	24.3 ± 11.3	31.9 ± 9.6	33.1 ± 8.6	0.1207	0.0005 (***)	0.5519
Pre SVA (mm)	44.3 ± 44.3	86.9 ± 35.3	38.4 ± 35.2	24.7 ± 35	0.0016 (**)	<0.0001 (****)	0.2461

**Table 3:** Comparison of operative details and the incidence of adverse events. (ASA—American Society of Anesthesiologists).

	ALL PATIENTS (*N* = 80)	TYPE 3 (*N* = 23)	TYPE 2 (*N* = 10)	TYPE 1 (*N* = 47)	TYPE 3 vs. 2	TYPE 3 vs. 1	TYPE 2 vs. 1
	Mean (±SD)				*p*-Value		
Operative time (min)	96.9 ± 45.9	108 ± 46.6	97.8 ± 63.4	91.4 ± 41.3	0.9361	0.4866	0.9779
Blood loss (cc)	229.6 ± 147.9	233.9 ± 175.1	185 ± 97.3	237 ± 143.4	0.8202	0.9998	0.7461
Hospital stay (days)	4.4 ± 2.9	4.7 ± 3.9	4.2 ± 1.5	4.4 ± 2.7	0.9635	0.9590	0.9986
Surgical Approach							
Anterior	4 (5%)	0	1 (10%)	3 (6.4%)			
Posterior	66 (82.5%)	20 (87%)	7 (70%)	39 (83%)			
Combined	10 (12.5%)	3 (13%)	2 (20%)	5 (10.6%)			
Intraoperative complications							
Incidental durotomy	2 (2.5%)	1 (4.3%)	0	1 (2.1%)			
High blood loss	0	0	0	0			
Hemodynamic instability	0	0	0	0			
Postoperative complications							
Within 30 days							
Early surgical revision	1 (1.25%)	0	0	1 (2.1%)			
Epidural hematoma	1 (1.25%)	0	0	1 (2.1%)			
Sepsis	1 (1.25%)	0	0	1 (2.1%)			
Screw/cage malposition	3 (3.75%)	0	0	3 (6.4%)			
ASA Score							
1	15 (18.75%)	1 (4.3%)	3 (30%)	11 (23.4%)			
2	30 (37.5%)	6 (26.1%)	3 (30%)	21 (44.7%)			
3	35 (43.75%)	16 (69.6%)	4 (40%)	15 (31.9%)			

**Table 4:** Comparison of clinical outcomes, satisfaction rate, and pain medication between MATCH and MISMATCH groups at one-year follow-up. Mann–Whitney *U* test was used to compare MATCH and MISMATCH variables. (*) correspond to *p* < 0.05.

	MATCH (*n* = 51)	MISMATCH (*n* = 29)	*p*-Value
ODI	18.9 ± 12.9	29.4 ± 18.9	0.0121 (*)
VAS Back (Scale 0–10)	4.2 ± 2.9	4.8 ± 3	0.4663
VAS Legs (Scale 0–10)	3.6 ± 3.1	3.8 ± 3	0.9018
Surgery Satisfaction score (%)	69.7 ± 20.7	62.6 ± 20.6	0.1611
Satisfaction			
Highly satisfied	15 (29.4%)	6 (20.7%)	
Satisfied	23 (45.1%)	17 (58.6%)	
No answer	3 (5.9%)	2 (6.9%)	
Unsatisfied	10 (19.6%)	3 (10.3%)	
Very unsatisfied	0	1 (3.5%)	
Pain medication	23 (45.1%)	14 (48.3%)	
Analgesic drug	15/23	10/14	
Non-steroidal anti-inflammatory drugs (NSAIDs)	3/23	2/14	
Both Analgesic + NSAIDs	2/23	1/14	
Opioids	3/23	1/14	

## Data Availability

The original contributions presented in the study are included in the article; further inquiries can be directed to the corresponding author.

## Supplementary Materials

The following supporting information can be downloaded at https://www.mdpi.com/article/10.3390/jcm14062041/s1: Table S1: One year post-operative Spinal Alignment Parameters following pre-operative subtype; Table S2: One year post-operative Spinal Alignment Parameters following pre-operative subtype and or MISMATCH groups.

## Abbreviations

The following abbreviations are used in this manuscript:

DSLS	Degenerative Spondylolisthesis of the Lumbar Spine
GG	Ghailane–Gille
ODI	Oswestry Disability Index
ASA	American Society of Anesthesiologists
HRQOL	Health-Related Quality of Life Scores
VAS	Visual Analog Scales
SL	Segmental Lordosis
LL	Lumbar Lordosis
PI	Pelvic Incidence
PT	Pelvic Tilt
SVA	Sagittal Vertical Axis
