# Supplementary material for: Does Adherence to Treatment Guidelines from the Ghailane–Gille Classification for Degenerative Spondylolisthesis of the Lumbar Spine Impact Surgical Outcomes? A Match–Mismatch Study
Source: J Clin Med. 2025 Mar 17;14(6):2041. doi: 10.3390/jcm14062041 (PMC11943325; doi:10.3390/jcm14062041)
Supplement: Supplementary file 1 [file jcm-14-02041-s001.zip › jcm-3467376-supplementary.pdf]

Supplementary Material

Table S1. One year post-operative Spinal Alignment Parameters following pre-operative subtype. Abbreviations: PT: Pelvic Tilt; PI: Pelvic Incidence; LL: Lumbar Lordosis; SL: Segmental Lordosis; SVA: Sagittal Vertical Axis.

**1 YFU**	All patients (n=80)	TYPE 3 (n=22)	TYPE 2 (n=10)	TYPE 1 (n=47)	Type 3 vs 2	Type 3 vs 1	Type 2 vs 1
	**Mean (± SD)**				**p Value**		
Pre PT (°)	22.38 ± 7.9	27.1 ± 8.5	22.5 ± 6.5	20.2 ± 7.1	0.9817	**0.0018 (**)**	>0.9999
Pre PI (°)	59.4 ± 11.4	65.8 ± 13.2	60.1 ± 11.9	56.1 ± 8.9	>0.9999	**0.0034 (**)**	0.8394
Pre PI-LL (°)	6.5 ± 13.4	16 ± 15.4	4.1 ± 10.5	2.5 ± 10.6	0.3612	**0.0010 (**)**	>0.9999
Pre SL (°)	9.6 ± 5.8	8.8 ± 4.9	6.4 ± 5.1	10.6 ± 6	>0.9999	>0.9999	0.1959
Pre LL (°)	59.5 ± 14.5	53.5 ± 17.6	65.8 ± 13.3	61 ± 12.3	0.2242	0.6449	>0.9999
Pre L4S1 (°)	24.8 ± 8.7	20.8 ± 11.3	27 ± 8.4	26.2 ± 6.6	0.3353	0.1783	>0.9999
Pre SVA (mm)	38.2 ± 32.7	58.5 ± 36.5	42.1 ± 37.1	27.9 ± 25	>0.9999	**0.0039 (**)**	0.7010

Table S2. One year post-operative Spinal Alignment Parameters following pre-operative subtype and MATCH (green case; n=50) or MISMATCH (red; n=28) groups are shown.

	Type 3 (n=22)	Type 2 (n=10)	Type 1 (n=47)	Type 3 MATCH vs Type 3 MISMATCH	Type 2 MATCH vs Type 2 MISMATCH	Type 1 MATCH vs Type 1 MISMATCH
	**Mean (± SD)**			**p Value**		
PT (°)	17.5 ± 7	22,1 ± 3.8	19.9 ± 6.8	**0.0043 (**)**	0.7619	0.6705
PT (°)	29.9 ± 6,8	22,9± 8.2	22.6 ± 9.7			
PI (°)	58.4 ± 15.5	60.6 ± 14.8	56.2 ± 9	0.1196	0.9143	0.8226
PI (°)	67.9 ± 12.1	61.2 ± 11.1	55.2 ± 9.6			
PI-LL (°)	5.9 ± 4.1	0.7 ± 9.9	1.7 ± 9.6	**0.0399 (*)**	0.3524	0.3864
PI-LL (°)	19 ± 16.4	6.3 ± 11.2	9.6 ± 16.6			
SL (°)	10.22 ± 3.6	6.6 ± 6.5	11 ± 6.1	0.3225	0.9143	0.4233
SL (°)	8.4 ± 5.3	6.2 ± 4.6	7.5 ± 5.2			
LL (°)	49.8 ± 8.9	72 ± 9	62.1 ± 11.8	0.4330	0.2571	0.1643
LL (°)	54.6 ± 19.5	61.7 ± 14.7	52.6 ± 14.9			
L4S1 (°)	23.4 ± 4.4	26.8 ± 7.5	26.7± 6.4	0.6588	0.9143	0.2953
L4S1 (°)	20 ± 12.6	27.1 ± 9.7	22.2 ± 8.1			
SVA (mm)	26 ± 19.3	29.3 ± 43.6	25.6 ± 23.8	**0.0082 (**)**	0.4762	0.1166
SVA (mm)	68.1 ± 35	50.7 ± 33.5	48 ± 28.7			
